# Family Caregiving for Persons With Emotional, Developmental, and Behavioral Disorders During COVID-19

**DOI:** 10.1093/geroni/igab046.1742

**Published:** 2021-12-17

**Authors:** Scott Beach, Richard Schulz, Heidi Donovan, Ann-Marie Rosland, Tara Klinedinst, Hansol Kim

**Affiliations:** 1 University of Pittsburgh, University of Pittsburgh, Pennsylvania, United States; 2 University of Pittsburgh, Pittsburgh, Pennsylvania, United States

## Abstract

COVID-19 has the potential to exacerbate stresses of family caregiving. While studies during COVID-19 have focused on caregivers of those with memory / cognitive issues like Alzheimer’s Disease, less attention has been paid to those caring for persons with emotional / developmental / behavioral disorders. This paper compares family caregivers of persons with emotional / developmental / behavioral disorders (EDB); physical conditions (PH); and memory / cognitive problems (MC) during the early phase of the pandemic. We focus on demographics, caregiving context, COVID-related caregiver stressors, and validated physical, psychosocial, and financial well-being outcomes. We conducted a cross-sectional national online survey during April-May, 2020 (n = 556). The sample included 274 PH (50%), 141 MC (25%), and 141 EDB (25%) caregivers. EDB caregivers were younger, with younger care recipients who were more likely to be their child. EDB caregivers reported more COVID-related caregiver stressors than MC or PH caregivers, including increased caregiving duties (p<.01); more family disagreements about care provision (p=.05); and worsened mental health of the care recipient (p<.01). In multivariate regression models, EDB caregivers had significantly higher anxiety; depression; and fatigue (all p<.01); more sleep disturbance (p=.05); less social participation (p<.05); and poorer overall financial well-being (p<.05). MC caregivers also reported more negative outcomes, but effects were consistently strongest for EDB caregivers. This study shows that EDB caregivers are at significantly elevated risk for negative impacts due to COVID-19 and should receive increased support and attention during this public health crisis.

